# State of the industry: integration of 3Rs principles and new approach methodologies in non-clinical product development through fit-for-purpose

**DOI:** 10.3389/fmed.2026.1887169

**Published:** 2026-08-26

**Authors:** Sabine M. Poppenborg, Debra Aub Webster, Björn Carlsson

**Affiliations:** SSI Strategy, Morristown, NJ, United States

**Keywords:** 3Rs principles, context of use, fit-for-purpose, new approach methodologies, non-clinical drug development, weight-of-evidence

## Abstract

Regulators, such as the FDA and EMA, are increasingly signaling expectations for broader and earlier adoption of New Approach Methodologies (NAMs) to reduce, refine, and, where justified, replace animal studies (3Rs principle)—particularly where NAMs can deliver improved human relevance, clearer mechanistic insight, or more efficient regulatory decision-making. This creates both an opportunity and a responsibility for drug developers: NAMs should no longer be viewed solely as exploratory tools, but as strategic components of an integrated pivotal non-clinical data package. This is already enshrined in various regulatory guidelines, roadmaps and position papers, but there is no single “approved list” of NAMs for pharmaceutical safety and efficacy assessment that applies across all products and agencies. Regulators explicitly support NAM adoption when scientifically sound, qualified for a defined context of use (COU), and integrated under a weight-of-evidence (WoE) approach. To support drug developers in responding to these expectations, a stepwise approach to expand the use of scientifically validated NAMs, such as *in vitro* human-based systems (human-organ-on-chip/ microphysiological systems, complex multicellular models, organoids, other 3D systems, and high-throughput screening), *in silico* methods (e.g., physiologically based pharmacokinetic (PBPK) modeling, machine learning, and computer modeling), and other innovative technologies is provided by authorities. This stepwise approach translates regulatory principles into an actionable pathway for qualifying, implementing, and defending NAMs in safety assessment. Such an approach should define the intended decision and COU, establish pre-specified acceptance criteria and performance expectations, integrate outputs under a transparent WoE framework, and incorporate early health authority engagement to de-risk adoption. The objective is not to promote NAMs as a universal substitute for animal studies but to provide a credible, regulator-aligned route to deploy NAMs where they can meaningfully improve decision quality while reducing animal use. Regulators recommend positioning NAMs as question-driven tools with clearly defined roles, enabling replacement, where performance and regulatory confidence are established, and reduction/refinement, where they optimize study design, dose selection, endpoints, and study sequencing.

## 1 Introduction

New approach methodologies include a variety of methods such as *in vitro* human cell systems, *in chemico*, and *in silico* models (e.g., patient-derived 3D/organoids, organs-on-a-chip/microphysiological systems [MPS], and computational modeling) that can reduce reliance on animals and have the potential to improve non-clinical to clinical translation (1, 2). NAMs are defined as alternatives to traditional animal tests (3, 4) and refer to novel methods that are compliant with the so-called 3Rs principles for the ethical use of animals in medicine testing across the European Union (EU). They represent potential alternatives to animal testing in the non-clinical development phase of new medicines (3). Health authorities emphasize that validity and reliability must be established before regulatory use (4).

The use of NAMs in drug development is not new to the pharmaceutical industry; there is an existing and established history of the routine use of validated methods in non-clinical development. A recently published article by the FDA Center for Drug Evaluation and Research (CDER) analyzed the landscape of studies using NAMs that were submitted to the FDA over the past 15 years in Module 4 of the Electronic Common Technical Document (eCTD) across various drug applications. The analysis focused on five NAM categories: *in vitro*, *in silico*, *in chemico*, non-human *in vivo*, and “other.” Results indicated that 93% of total NAM submissions were concentrated on two categories: *in silico* (49%) and *in vitro* (44%). *In vitro* NAMs, including stem cell-derived models, sandwich culture models, and co-culture models, showed higher prevalence compared to 3D models, reconstructed tissue models, and human organ chip/MPS models. Significantly more *in vitro* NAMs were submitted in investigational new drug (IND) applications compared to new drug applications (NDA) and Biologics License Application (BLA) applications. The authors emphasized that the analysis did not evaluate the COU, methods, endpoints, or conclusions from the models used in the study reports (5).

The applicability of NAMs in drug development and their regulatory acceptance is dependent on the drug modality and the availability of established non-clinical models. In cases where no scientifically relevant animal species/exogenous target exists, the regulatory threshold for the use of alternative or novel *in vitro* methodologies is reduced. These models are more readily accepted to support non-clinical decision-making when their COU and scientific validity are adequately supported. NAMs may be particularly useful in the characterization of new modalities with respect to safety signals that may not be seen in animals due to species specificity. In this case, human-based NAMs may be particularly relevant not only with respect to safety but also for pharmacodynamic (PD) translation.

The use of *in silico* models is becoming increasingly important with respect to data integration, analysis, and the potential to predict toxicological and pharmacological endpoints. Examples of *in silico* models include

- PBPK modeling: Models how chemicals are absorbed, distributed, metabolized, and excreted in the body
- Quantitative structure–activity relationship (QSAR) models: Prediction of a chemical’s activity based on its structure
- Quantitative systems pharmacology (QSP): Integration of complex, disease-specific biological data with pharmacological principles to simulate how a drug will alter disease progression
- Machine learning/artificial intelligence to simulate biological processes, optimize drug design, and replace or reduce animal testing

Well-validated *in silico* models therefore play a critical role in enabling structured data interpretation, supporting decision-making, and enhancing the translational and regulatory value of complex non-clinical datasets (5).

The integration of NAMs is now well established in the early discovery phase of drug development, where they are applied to support clinical candidate selection, build confidence in assay performance, and enable mechanistic and PD characterization. In the safety domain, however, NAMs can already support replacement or reduction of animal use in defined, scientifically validated contexts, but they do not yet provide a general substitute for *in vivo* studies across all endpoints; chronic/repeat-dose toxicity remains a particular limitation under the current regulatory framework (3, 6, 7). The biological limitations, such as lack of systemic physiology, immune-component, or endocrine integration, remain despite the increasing specificity of modern drug development programs, such as targeted therapies, which generally reduce off-target toxicity compared with traditional small-molecule approaches. Additionally, technical limitations associated with donor-to-donor variability, assay reproducibility, or protocol variability must be acknowledged and addressed in their implementation. However, there is confidence that modeling more complex multi-organ physiological and toxicity responses remains a real possibility (6), and NAMs will play an important complementary role in risk assessment and mitigation. For example, when liver safety signals emerge during clinical development, established *in vitro* NAMs for drug-induced liver injury (DILI) can be strategically deployed to investigate mechanisms, de-risk the program, and inform mitigation strategies (6).

It should be noted that there are several NAMs that are qualified within a given COU, such that the results of these assays can be relied on to have a specific interpretation and used in regulatory decision-making. These include, for example, the *in vitro* secondary pharmacology panels (off-target receptor binding), human ether-a-go-go-related gene (hERG)/cardiac ion channel assay (International Council for Harmonisation of Technical Requirements for Pharmaceuticals for Human Use (ICH) S7B) (8), *in vitro* Ames assay (ICH S2(R1)) (9), *in vitro* mammalian cell micronucleus test (ICH S2(R1)) (9), wavelength absorption (*in vitro* photosafety testing, ICH S10) (10), and *in silico* evaluation of impurities (ICH M7(R2)) (11).

NAMs also provide high value in early safety de-risking, DDI/human exposure prediction, and mechanistic investigations that reduce iterative *in vivo* cycles. PBPK and *in vitro* ADME/safety profiling can materially improve dose selection and reduce exploratory animal work. Regulators explicitly recognize that integrating advanced *in vitro* systems with computational modeling (including PBPK) can reduce or replace animal use: FDA notes that, “to minimize animal testing,” an integrative approach can, for example, combine a “human organ chip for toxicity” with “a PBPK model” for PK, and an artificial intelligence immunogenicity predictor (7), while EMA states that incorporating NAMs into non-clinical assessment “could replace or reduce animal use” by, for example, “*in vitro* (cell-based) systems and computer modeling” as examples (12).

There is overlapping and sometimes inconsistently used terminology in the field—particularly around validation, qualification, and fit-for-purpose—all of which lead to regulatory acceptance. Validation is specifically defined by FDA as related to the accuracy, reliability, and relevance of a NAM; it establishes confidence in the quality of the data from the NAM and is critical in establishing reliability. Validation considerations include context of use, human biological relevance, technical characterization, and fit-for-purpose. Qualification is a regulatory determination about how a NAM may be used and is addressed through separate FDA programs and guidance (13). EMA has a similar program: Qualification of Novel Methodologies (14). Once a NAM is qualified for the intended context of use through these programs, the NAM is broadly accepted to support regulatory decision-making. These concepts are further discussed in Section 5. A set of working definitions used throughout this manuscript is provided in Table 1. These definitions are aligned with current FDA and EMA terminology but are structured to support practical implementation.

**Table 1:** Definitions of key terms and illustrative regulatory case examples for the implementation of new approach methodologies in non-clinical drug development.

Term	Definition (regulatory context)	Regulatory consequence	Case example
Context of Use (COU)	A full, clear, and concise statement describing the specific regulatory purpose a method is intended to address, including the decision it informs, the conditions under which it is applied, and its limitations.	Determines the level of evidence and validation required and whether the method/tool is appropriate for the intended regulatory purpose.	Use of a human Liver Chip to assess drug-induced liver injury (DILI) risk in early clinical candidate selection (6, 38). Biomarker panel for predicting drug-induced kidney injury (DIKI) (43).
Fit-for-Purpose	The principle that a method should have sufficient reliability, relevance, and performance characteristics to support a specific decision within its defined COU.	Determines the acceptability of a method for regulatory use and its suitability for regulatory decision-making (e.g., exploratory vs. pivotal support).	An *in vitro* potency assay used to support dose selection, without replacing all toxicology studies (Table 2).
Validation	The structured process of establishing that a method is accurate and reliable (reproducible across labs) and relevant for a defined purpose.	Supports scientific credibility but does not by itself guarantee regulatory acceptance.	OECD test guideline development for an alternative skin sensitization assay (44); biomarker panel for predicting DIKI (43).
Qualification	A formal regulatory process (e.g., FDA DDT/ISTAND, EMA Qualification of Novel Methodologies) whereby a validated method is accepted for a specific COU based on submitted evidence.	Enables broader regulatory use across programs for the qualified COU without the need for re-justification in each submission.	Qualification of a biomarker panel for predicting DIKI (43).
Regulatory Acceptance (General)	The extent to which a regulator is willing to consider and rely on data from a method in decision-making, which may be case-by-case or formalized (EMA: case-by-case or via Scientific Advice; FDA: via IND/NDA submission or ISTAND).	May range from informal acceptance in a single submission to formal qualification; not all accepted methods are formally qualified.	Inclusion of *in silico* PBPK modeling to support labeling or dose adjustments in a specific application (Table 2).
Weight-of-Evidence (WoE) /Integrated Approach to Testing and Assessment (IATA)	An approach or structured framework that integrates multiple data sources (e.g., *in vitro*, *in silico*, *in vivo*, clinical) to support a regulatory conclusion/ decision-making, considering strengths and uncertainties of each component.	Enables combined use of NAMs and traditional data, particularly when no single method is sufficient.	Combining organ-on-chip data, PBPK modeling, and limited animal data to assess human safety risk (7); IATA for skin sensitization combining *in chemico*, *in vitro*, and computational methods (44).
Replacement / Reduction / Refinement (3Rs)	Ethical and scientific framework to replace animal use where possible, reduce the number of animals used, and refine methods to minimize suffering.	Guides policy direction and regulatory innovation but does not override evidentiary requirements for safety.	Replacing an animal study with a validated *in vitro* assay (replacement) or reducing group sizes using model-informed approaches (reduction), or refining study design by using NAM-derived data to optimize dose selection (refinement) (7, 12, 30).
Case-by-Case Acceptance	Use of a NAM within a specific regulatory submission, supported by sponsor-provided justification, without prior formal qualification. Based on scientific validity.	Common pathway for early adoption; requires justification each time.	Use of a novel organoid assay to support mechanistic understanding in a single IND (Table 2).
Regulatory Qualification vs. Application-Specific Use	Distinction between methods that are formally qualified for a defined COU and those used *ad hoc* in individual submissions.	Clarifies whether a method has broad regulatory standing or requires repeated justification.	A qualified safety biomarker vs. a sponsor-specific *in vitro* assay used once (43).

COU, context of use; DDT, drug development tool; DILI, drug-induced liver injury; DIKI, drug-induced kidney injury; EMA, European Medicines Agency; FDA, Food and Drug Administration; IATA, integrated approach to testing and assessment; ICH, International Council for Harmonisation; IND, investigational new drug application; ISTAND, innovative science and technology approaches for new drugs; NAM, new approach methodology; OECD, Organisation for Economic Co-operation and Development; PBPK, physiologically based pharmacokinetics; vs, versus; WoE, weight of evidence; 3Rs, replacement, reduction, refinement.

## 2 NAM implementation

Recently, the FDA has released a draft guidance (13) to facilitate broader integration of scientifically validated NAMs in drug development and regulatory submission packages and in alignment with its *Roadmap to Reducing Animal Testing in Preclinical Safety Studies* (7) to improve predictive toxicology in humans. The translation of human-relevant, mechanistic non-clinical data into evidence that can enable regulatory decision-making requires that the NAM be implemented within a disciplined, regulatory-aligned framework rather than as *ad hoc* scientific enhancements. This requires consideration of (i) clearly defined COU and fit-for-purpose evidence, (ii) integration under a WoE approach, (iii) quantitative translation to human exposure, (iv) proactive health authority alignment and endorsement/acceptance, and (v) 3Rs-by-design. Without clear articulation of the COU and fit-for-purpose intent, NAMs risk being considered exploratory, limiting their influence on regulatory conclusions. With thoughtful implementation, NAMs can move beyond supportive mechanistic tools to become credible contributors to regulatory confidence, enabling informed decision-making across development milestones without compromising safety assurance (3, 4, 12, 14–16). Operationalizing NAM integration requires anchoring the non-clinical strategy to the target product profile (TPP) with respect to clearly articulating the COU and what evidentiary needs the NAM is intended to provide. Based on cumulative guidance from health authorities, the following considerations are central to determining the appropriateness and regulatory value of NAM incorporation:

- Product modality (e.g., small molecules, biologic, biosimilar, advanced therapy medicinal products [ATMP]): Are there modality-specific attributes, such as potential off-target pharmacology, immunogenicity, or human-specific biology that NAMs may address?
- Indication/patient population: The acceptance of NAMs in non-clinical evidence generation will be influenced by whether the product is intended to address, e.g., an unmet medical need, a rare disease, use in pediatric populations, and the severity of the disease.
- Clinical translation: Deployment of NAM PD assessments to inform clinical dose selection will require demonstration of relevance of the PD biomarker measured as predictive of efficacy.
- Mode-of-action (MoA) and underlying biology: NAMs may offer superior human relevance compared with traditional animal models for products with a human-specific MoA, potential for immune reaction, or species specificity.
- Manufacturing maturity: NAMs may offer optionality with respect to evaluation of process stability and product comparability.
- Regulatory pathway (accelerated vs traditional vs generic/biosimilar): Will data from a NAM meet the evidentiary expectations of the non-clinical package for the intended regulatory pathway?
- Product development plan: Deployment of a NAM should be considered in the context of the overall product development plan and whether NAMs are intended to inform, supplement, or replace/reduce/refine specific animal studies, and acknowledgment of acceptable residual uncertainty.

### 2.1 Product modality considerations

For biologic modalities, species specificity frequently constrains the identification of a pharmacologically relevant animal model and may, in some cases, preclude the application of conventional toxicity study designs. In parallel, immunogenicity is explicitly acknowledged as an intrinsic property of biologics that can confound the interpretation of non-clinical findings and limit their direct translational value; accordingly, *in vitro* assays (e.g., target binding and cell-based potency) and functional assays (species-specific cell-based systems and tissue cross-reactivity) are recommended to characterize pharmacology, quantify human versus animal sensitivity, and support extrapolation to humans (ICH S6(R2) (17)).

For biosimilar development, regulators explicitly endorse a stepwise, totality-of-evidence development paradigm in which residual uncertainty is progressively reduced through analytical, structural, and functional comparability, with any additional *in vivo* studies justified solely by the specific uncertainties that remain following these assessments (18–21). The core message from regulators is that *in vitro* methods and *in silico* approaches (e.g., comparative *in vitro* functional and potency assays to define MoA, binding assays and effector function assays, human primary-cell assays, cross-species target binding or cross-reactivity, *in silico* pharmacokinetics (PK)/exposure comparison, cytokine release assay, and epitope mapping) are regarded as more sensitive, specific, and scientifically informative than animal studies for identifying meaningful differences between a biosimilar and its reference product. These methods are therefore regarded as the primary drivers of biosimilarity assessment, with animal studies playing only a limited, confirmatory role, where this is justified by a clearly defined residual risk (20).

For gene and cell therapy products, NAMs frequently move from a “supportive” role to a program-enabling pillar of the non-clinical strategy, reflecting the limited predictivity of conventional animal models in the context of pronounced species specificity, divergent immune responses, and complex biodistribution and persistence profiles (22, 23). Accordingly, the recommended development paradigm emphasizes NAM-led mechanistic characterization (potency, specificity, immune risk, and tissue liabilities), followed by targeted *in vivo* studies designed to address clearly defined residual uncertainties and to support clinical monitoring plans. Regulatory guidance for ATMPs increasingly favors a mechanism and human-relevance-first (human-centric), NAM-driven non-clinical framework that includes human biology and uses advanced *in vitro* and computational evidence to characterize pharmacological activity, immune risk, tissue liabilities, translation to first-in-human (FIH) (such as biodistribution, persistence, and shedding), and clinically monitorable parameters. Within this framework, biologically justified *in vivo* studies are positioned as confirmatory and risk-focused, intended solely to resolve remaining uncertainties (13, 24, 25).

### 2.2 Indication/patient population considerations

Indication- and population-specific factors—including the degree of unmet medical need, disease severity, rare disease context, and pediatric populations—directly influence risk tolerance and the appropriate balance between innovation and conservatism in non-clinical evidence generation. These considerations are central to defining both the scope and the regulatory acceptability of NAM integration.

Rare disease development in particular amplifies the same strategic intent—use NAMs as question-driven tools inside a WoE framework; however, the execution must account for limited patient numbers, constrained clinical tolerance for uncertainty, and frequent gaps in disease natural history and use of biomarkers. Since rare diseases commonly involve unique biology, limited predictive animal models, and high unmet need, NAMs should be front-loaded to generate human-relevant evidence, deliberately deploying human-relevant, non-animal experimental, and computational tools, such as patient-derived cells and organoids, induced pluripotent stem cell (iPSC) models, microphysiological organ-on-chip systems, and integrated omics platforms, at the earliest possible stages of drug discovery and development, rather than treating them as late-stage confirmatory studies. In the examples provided in Table 2, a patient-specific iPSC–cardiomyocyte platform provided a translational bridge linking the molecular mechanism of action—elamipretide’s effect on mitochondrial cardiolipin—to a clinically meaningful intermediate endpoint: knee extensor muscle strength. This continuity from subcellular target to functional patient outcome exemplifies how a front-loaded NAM can carry mechanistic confidence across the full translational chain. In selected cases, NAM approaches—such as disease-relevant *in vitro* systems, *ex vivo* tissue explants, or participant-derived cellular models—can provide early proof-of-concept, mechanistic validation, and translational confidence such that any remaining animal studies would be minimized, targeted, and directly tied to FIH dose selection, escalation, and patient safety monitoring (26).

**Table 2:** NAM case examples: purpose/readout and regulatory positioning.

NAM model	Purpose/Functional read out	Regulatory framing	Reference
Human intestinal organoids derived from cystic fibrosis (CF) patients	CF transmembrane conductance regulatory (CFTR) activity quantified via organoid swelling (a direct measure of chloride transport restoration)	Mechanistic/functional support and translational bridging to clinic	EMA, EPAR, 2018 Orkambi (lumacaftor/ivacaftor) (45)
Molecular assays for vector biodistribution/persistence and transgene expression (qPCR, RT-qPCR)	Vector DNA (qPCR) and vector-derived mRNA (RT-qPCR) in relevant matrices / tissues	All assays were fit to purpose and validated according to recent guidance ICH S12.	EMA, EPAR, 2023 Hemgenix, etranacogene dezaparvovec for gene therapy (46)
Induced pluripotent stem cell (iPSC)-derived cardiomyocytes from subjects with Barth syndrome (BTHS), an ultra-rare disease	To model human disease-specific mitochondrial and cardiomyocyte dysfunction *in vitro*	Mechanistic/functional support; translational bridge linking mechanism of action (on mitochondrial cardiolipin) with clinical endpoints (knee extensor muscle strength assessed by handheld dynamometry).	FDA, NDA 215244, 2025 Forzinity (elamipretide) (47)
*In vitro* human cell assays: co-culture studies using bone marrow-derived culture-expanded mesenchymal stem cells (MSCs) with allogeneic immune cells	Suppression of alloreactive T-cell proliferation, modification of cytokine secretion profiles of immune cells in culture	Supportive pharmacology/ PD evidence consistent with the proposed immunomodulatory mode-of-action, alongside clinical PD biomarker	FDA, SBRA, 2024, STN: BLA 125706/0 RYONCIL (remestemcel-L-rknd), 2024 (48, 49)
*In vitro* human primary CD34 + hematopoietic stem and progenitor cells (HSPCs) from healthy donors and patients edited with CRISPR/Cas9 (SPY101-RNP)	Establish mechanistic pharmacology and proof of function; hybrid-capture confirmatory testing: potential off-target, unintended genome editing	On-target editing efficiency and basis for risk assessment	FDA, SBRA, 2024; EMA, EPAR 2024 Casgevy (exagamglogene autotemcel; CRISPR-edited autologous CD34 + cells) (50, 51)
*In silico* simulator / Simcyp v19 PBPK platform	To predict the magnitude of CYP-mediated drug–drug interactions (DDIs)	Qualified for predicting the average magnitude of interactions mediated by CYP enzymes within the specific COU 1–3 as defined in the EMA Qualification Opinion.	EMA, Simcyp Simulator, EMA Qualification Opinion, 2025 (52)
*In silico* PBPK modeling approach	Quantitative exposure predictions and DDI risk (impact of CYP3A4 inhibitors/inducers)	Model-informed clinical pharmacology tool to support risk assessment and labeling	FDA, NDA: 217639, 2022, approved 2023 Orserdu, Elacestrant (53)

BLA, Biologics License Application; BTHS, Barth syndrome; CF, cystic fibrosis; CFTR, CF transmembrane conductance regulator; COU, context of use; CYP, cytochrome P450; DDI, drug–drug interaction; EMA, European Medicines Agency; EPAR, European Public Assessment Report; FDA, the Food and Drug Administration; HSPC, hematopoietic stem and progenitor cell; iPSC, induced pluripotent stem cell; MoA, mode-of-action; MSC, mesenchymal stem cell; PBPK, physiologically based pharmacokinetic; PD, pharmacodynamics; qPCR, quantitative polymerase chain reaction; RT-qPCR, real-time qPCR, NDA, New Drug Application; SBRA, Summary Basis for Regulatory Action; STN, submission tracking number.

### 2.3 Exposure consideration

Physiologically based pharmacokinetic modeling provides well-accepted data supporting clinical dose translation and decision-making (extrapolation of *in vitro* data) using mixed types of modeling (e.g., mechanistic modeling, machine learning, and *in silico* analysis). The quantitative foundation of PBPK modeling is built on high-quality non-clinical *in vitro* and *in vivo* data that enable exposure-response relationships. Pharmacokinetic inputs derived from human *in vitro* ADME systems—such as intrinsic clearance, transporter interactions, permeability, and plasma or tissue binding, enzyme kinetics, non-clinical *in vivo* PK data—provide the quantitative foundation for PBPK modeling. These data enable translation of *in vitro* concentration–response relationships into predicted human exposure, thereby supporting clinically relevant, exposure-driven decision-making in line with ICH M12 (27) and current EMA/FDA PBPK guidance (28, 29).

### 2.4 Biological considerations

NAMs should be based on biologically relevant systems that appropriately reflect human physiology for the scientific or regulatory question being addressed. This includes consideration of cell or tissue origin (human vs. animal), donor characteristics (age, sex, disease status, and genetic background), and the relevance of target expression and biological pathways. NAMs capture only selected aspects of human biology; therefore, it is essential to articulate which biological processes are represented and which are not, including the absence of systemic interactions (lack of whole-body integration), compensatory mechanisms, or chronic adaptation. Biological information from NAMs is most impactful when considered alongside orthogonal evidence from *in silico* models (e.g., PBPK modeling and QSP), *in vivo* studies, and clinical or translational data, supporting risk-based decision-making and, where appropriate, reduction or refinement of animal studies; consistent with EMA reflection papers on the implementation of the 3Rs and FDA roadmaps promoting scientifically validated NAMs (7, 12, 15, 21, 30).

Biological considerations are central to biomarker development, as NAMs enable the identification and qualification of mechanistically anchored, human-relevant biomarkers that reflect target engagement, pathway modulation, and downstream functional effects, thereby supporting translational exposure–response assessment and clinically actionable decision-making across early development.

### 2.5 Manufacturing considerations

Manufacturing maturity and control are critical determinants of the interpretability and regulatory utility of NAM-derived data dependent on the degree to which the tested product is representative of the clinical product in terms of quality attributes, formulation, and process-related variability. For both small molecules and biologics, early alignment between manufacturing and non-clinical strategies is essential to ensure that NAM studies are conducted with material of appropriate quality and comparability. Across modalities, regulators expect transparency regarding the manufacturing status of the test article used in NAM studies, including its relationship to the intended clinical material and any known or anticipated changes. In addition to the comparability of the product tested in a NAM assay to the clinical material, NAMs can also be deployed as a tool for evaluation of process stability and product comparability. Non-animal-based methods are routinely accepted as part of a potency assay matrix. FDA/EMA encourage sponsors to use these assays to develop non-animal-based potency assays and that *in vivo* (animal) assays should be avoided or replaced whenever scientifically feasible and adequately validated. Inclusion of these early in development could support eventual use as the sole potency release assay. EMA and FDA guidelines explicitly state that *in vitro*, mechanism-of-action–based potency assays should be the method of choice (31–33).

## 3 Regulatory considerations

It is important to consider how data from a NAM will meet the evidentiary expectations of the non-clinical package for the intended regulatory pathway. If a biomarker is to be used as an endpoint to support accelerated approval, NAMs may provide the PD data to link a biomarker to the clinical outcome it is intended to predict. NAMs may be more acceptable for generics or biosimilars, given availability of data on the predicate products. The recent FDA guidance outlining a streamlined approach to non-clinical study for monoclonal antibodies specifically mentions inclusion of assays assessing human-relevant off-tissue binding and potential secondary effects and NAMs as other non-clinical data to be included in a WoE approach to justify restricting chronic toxicity studies to 3 months (34).

Regulatory-compliant and qualified innovative research methodologies are particularly relevant to overcome the scarcity of data and the lack of validated endpoints and biomarkers in research fields characterized by small samples such as rare and pediatric disease (35).

While both FDA and EMA endorse the scientific validity and regulatory acceptance of NAMs, important procedural and evidentiary differences merit explicit discussion. The EMA has formalized a structured qualification opinion pathway with defined timelines and scientific review criteria, whereas the FDA has historically favored case-by-case acceptance within individual regulatory submissions, allowing more flexibility but requiring sponsors to justify scientific validity anew for each application. Additionally, the agencies differ in their evidentiary expectations regarding validation standards, the role of consensus guidance, and the timeline for implementation—factors that significantly impact development strategy and regulatory planning for sponsors pursuing global submissions.

## 4 Product development considerations

Product development considerations are also central to defining the strategic role and regulatory value of NAMs across the non-clinical program. Regulators increasingly expect the use of NAM to be explicitly contextualized within a WoE framework, with clear articulation of how NAM-derived data inform specific development decisions and how any residual uncertainty is managed. Both agencies (EMA and FDA) provide clear product-development “rules of engagement” for NAMs—centered on COU, fit-for-purpose evidence, WoE integration, and regulatory pathway and early regulator interaction—even though neither offers a single universal template.

## 5 Integration of NAMs into non-clinical strategy

**Figure 1:**
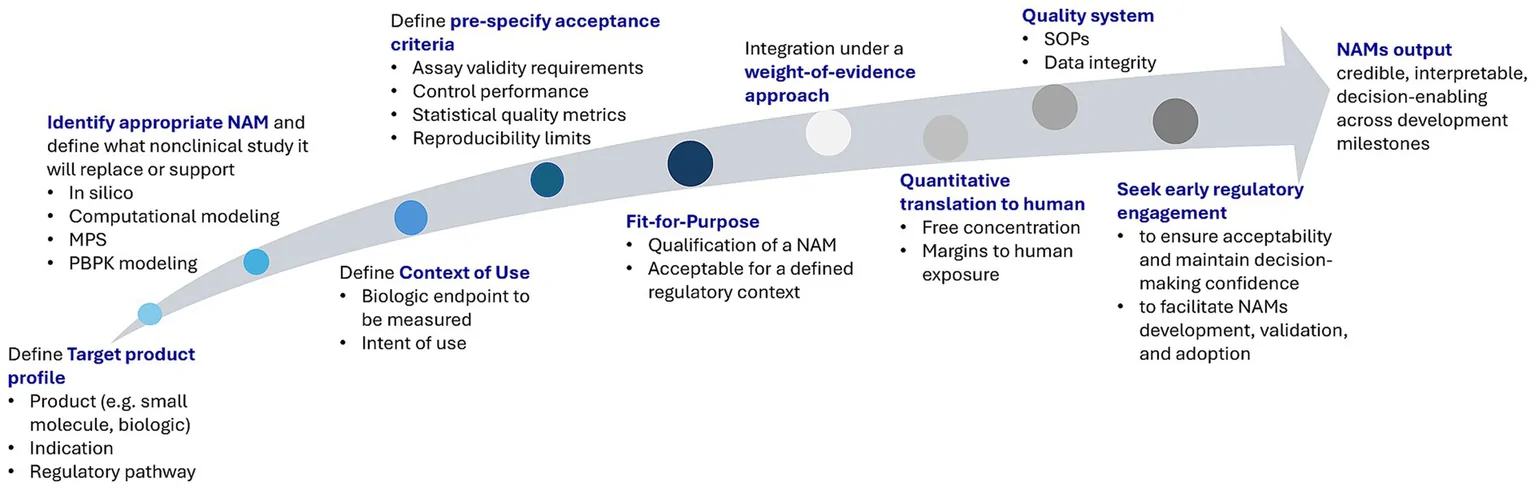
Strategic implementation of NAMs. Illustration of an implementation plan for a ‘New Approach Methodology’ (NAM) aimed at obtaining regulatory approval. MPS, microphysiological systems; PBPK, physiologically based pharmacokinetic modeling; SOP, standard operational procedures.

A pragmatic approach for integrating NAMs into the overall drug development strategy that is consistent with regulatory expectations and key decision points is shown in Figure 1.

The guiding concept is straightforward: NAMs should be integrated into the non-clinical development plan as decision-enabling tools rather than stand-alone experiments. Sponsors should begin by defining the specific regulatory decision the NAM is intended to support (e.g., study replacement vs. reduction/refinement vs. mechanistic support), then demonstrate that the NAM is fit-for-purpose within a clearly defined COU including its applicability domain, performance expectations, and limitations, and integration under a WoE approach. Finally, sponsors should make residual uncertainty explicit, quantify it where feasible, and translate it into a risk-based evidence and mitigation plan (e.g., orthogonal evidence, exposure translation, targeted *in vivo* only where necessary, and clinical risk controls) that can support early and purposeful regulatory engagement. This approach aligns with FDA’s stated view that NAMs can strengthen drug development when integrated with mechanistic understanding and other relevant evidence, and it operationalizes the principle of replacement, reduction, refinement (3Rs) through intentional program design (4, 7). FDA presents four key validation principles: COU, biological relevance, technical characterization, and fit-for-purpose, to ensure NAM studies are reliable and interpretable for regulatory use (13). Adequate demonstration of the biological relevance of a NAM provides confidence in the methodology.

### 5.1 Context of use

Central to the choice of a NAM (e.g., *in silico*, computational modeling, human organ-on-a-chip, advanced *in vitro* assays, MPS, PBPK modeling) is defining the COU. That is what non-clinical study will it support or replace, which specific question will it address (pre-specify acceptance criteria), and what role the assay plays in the integrated risk assessment as part of the WoE for use in regulatory decision-making; NAM utility depends on context, not technology alone (36). At a minimum, the COU should specify the regulatory question being addressed, the endpoint and associated risk, the relevant patient population and exposure range, and how the NAM output will be used to support decisions, including predefined thresholds and follow-up actions. This framing aligns with regulatory expectations that novel methodologies be justified for a specific intended use, rather than proposed as broadly applicable tools (3). When a NAM is used to replace an animal study or inform a high-impact decision (e.g., FIH dose selection, safety margins, or study waivers), the COU should also describe the decision risk and mitigation strategy, such as use of orthogonal evidence, conservative assumptions, or clinical monitoring triggers. The COU should guide both biological qualification and technical validation of the NAM.

### 5.2 Fit-for-purpose use: validation versus qualification

Key challenges in implementing NAMs include the complexity of biological and donor-derived starting materials, particularly in advanced systems such as MPS, and persistent reproducibility issues, including inter-laboratory variability. Both EMA and FDA emphasize validation of a NAM as a prerequisite to be formally adopted for regulatory acceptance. Validation in the context of NAMs is a process that establishes technical characterization, that is, accuracy (e.g., precision) and reliability (e.g., reproducibility), and relevance for a specific COU (ICH Q2(R2)) (37). While technical validation is independent of a specific regulatory decision, regulators must have confidence in the method with respect to the output range for biomarkers and the baseline of the model’s readout in order to be confident in the intended use of the NAM. Validation is necessary to determine the quality of the data generated and to support the health authority in regulatory decision-making (13). However, as indicated in the draft FDA guidance, even if not validated, a NAM may “adequately address specific toxicological concerns” (13). In this case, assays must be validated to a level appropriate for their intended regulatory purpose; again, they must be “fit-for-purpose.” Whether validated or fit-for-purpose, NAMs are always used in the context of the WoE provided by prior knowledge and preclinical toxicology information. A fit-for-purpose NAM can support drug development by replacing or improving traditional methods, addressing data gaps where conventional models are insufficient, and confirming or complementing existing findings.

In contrast, qualification determines whether a validated NAM, considered as a drug development tool (DDT) (13, 38) and its proposed COU, is acceptable for a defined regulatory context, such as supporting the waiver of an *in vivo* study or could also contribute to a reduction in animal testing (7, 12, 15, 21, 30). The qualification of a NAM is generally handled by regulators as the qualification of a DDT (13, 38) or qualification of novel methodologies/biomarkers (14, 39, 40). The “fit-for-purpose” qualification process includes definition of COU, demonstration of analytical reliability, biological relevance, and confirmation of regulatory qualification. Qualification is about establishing confidence that a technically validated NAM can reliably support a specific regulatory decision; for example, a drug testing positive in wavelength absorption (*in vitro* photosafety testing, ICH S10 (10)) will invariably be reflected as phototoxic potential in product labeling.

### 5.3 Regulatory acceptance

The road to regulatory acceptance of a NAM is discussed in numerous documents and guidance issued by EMA and FDA. EMA’s regulatory acceptance principles indicate that to be considered as alternatives to animal tests, NAMs must be scientifically sound to be useful in regulatory decision-making, which implies formal validation of method performance (3, 12). FDA’s *Roadmap to Reducing Animal Testing in Preclinical Safety Studies* and new draft guidance repeatedly use the term “scientifically validated NAMs,” indicating that NAMs must be validated with regard to reliability and confidence in the methodology before regulators will consider them as alternatives to animal tests (7, 13). Both agencies stress the importance of engagement and discussion of NAM integration into the IND and clinical trial application (CTA), enabling a non-clinical development plan.

Sponsors should plan early regulatory engagement (as early as possible) to align on the scientific question the NAM is intended to answer, how the data generated can be used in regulatory decision-making, and what is the confidence the data the NAM generates is reproducible and trustworthy. Both EMA and FDA indicate that acceptance of alternative methods requires continuous dialog and feedback between all partners from development to implementation. EMA provides established routes for discussing innovative methodologies and 3Rs implementation, including Innovation Task Force (ITF) briefing meetings for early dialog on NAM readiness and feasibility, and Scientific Advice to align on the proposed role of NAMs in the development program and the evidence needed to support that role (3, 15, 21, 41). To facilitate development of 3R testing approaches, EMA encourages sponsors to share NAM data via Voluntary Data Submission (VDS). The objective of the VDS is to familiarize regulators with emerging NAMs and allow sponsors to obtain early scientific feedback without regulatory risk to ongoing or future development programs (3, 21). The EMA Qualification Opinion (a formal, scientific assessment by the Committee for Medicinal Products for Human Use [CHMP]) provides a public, regulator-endorsed COU for a methodology, making expectations transparent and enabling broader sponsor confidence in use. Broader NAM regulatory landscape and horizon scanning are actively being addressed by EMA via dedicated materials highlighting regulatory considerations and acceptance pathways (30, 41).

The FDA ISTAND program allows for full qualification of the NAM as a DDT. Multiple NAMs are currently under review under this program and recently, a Human Liver-Chip (organ-on-a-chip) technology for Prediction of Drug-Induced Liver Injury was accepted into the program as a DDT “*used to assess the risk of small molecule candidate drugs inducing liver injury in adults to create human-relevant data for the candidate drug’s IND application, providing greater evidence of safety assurance for regulatory decision making that enables Phase I clinical trials*” (6, 38). Once qualified, the DDT can be used for a defined COU, and the DDT for that defined COU can then be used by any sponsor (not just the original submitter). Additionally, questions regarding NAM assay development can be addressed in a CDER NAMS coordinating meeting. To facilitate discussion with FDA, sponsors should consider what questions may be asked by regulatory authorities in evaluating COU and fit-for-purpose with respect to both NAM use qualification and NAM analytical validation, for example:

- What decision does this data support?

Will they be used to support exposure translation linking hazard signals to human exposure scenarios (often PBPK/model-informed) or to replace an *in vivo* study that evaluates a certain toxicity? NAMs will be interpreted in the context of WoE integration, that is, interpreted alongside existing knowledge (platform data, literature, *in vivo* where needed), and an understanding of residual uncertainty is critical.

- What would happen if the assay were wrong?

Regulators will evaluate assay acceptability by asking whether incorrect results would lead to unacceptable downstream consequences – and whether those consequences are mitigated by validation, decision design, or safeguards. Sponsor must be prepared to address how uncertainties in the assay performance are mitigated in the overall development strategy.

- Are uncertainties transparently described?

What is the acceptable level of residual uncertainty for the indication (oncology vs. chronic disease vs. pediatrics/rare)? FDA and EMA frame acceptability as context-dependent, driven by benefit/risk, unmet need, and the consequences of being wrong. It is expected that uncertainty should be actively managed and mitigated by use of orthogonal assays, conservative exposure assumptions, monitoring, and/or postmarketing commitments.

## 6 State of the industry and future directions

NAMs are now routinely used across discovery and early development, particularly for mechanism confirmation, target and pathway engagement, high-content phenotyping, early safety de-risking, drug–drug interaction prediction (e.g., PBPK), and translational biomarker strategy. Examples of NAMs used as mechanistic/functional evidence in regulatory submissions (purpose, evaluations, and regulatory framework) are shown in Table 2. Case examples have been selected based on the following: (i) examples of different categories (*In vitro* human cell assays, in silico), (ii) documents submitted to both health authorities (the EMA and the FDA), which accepted NAMs in the marketing authorizations were reviewed, in order to demonstrate that the use of NAMs is accepted by both health authorities, and (iii) addressing different regulatory framing.

Due to persistent limitations in the predictive accuracy of certain animal models, evolving ethical and societal expectations, advances in stem-cell and microphysiological technologies, and increased regulatory support for the integration of NAMs within well-defined contexts, these approaches are becoming more widely adopted for toxicological testing and mechanistic research (1, 2). At the same time, the industry is not yet at a point where animal studies can be broadly replaced across the non-clinical safety package. The primary constraint is not scientific ambition but regulatory and operational reality: NAMs are highly capable for certain questions, but they do not consistently reproduce the full systemic complexity that regulators rely on for risk characterization—multi-organ interactions, immune and endocrine contributions, adaptive responses over time, and idiosyncratic toxicities (2). Shenton and coworkers report that NAM-based regulatory submissions—particularly for biotherapeutics—have been generally accepted by global health authorities when the justification is clear (e.g., no pharmacologically relevant species, prior experience with the target, and/or high disease severity/urgent benefit/risk context) (1). A 2026 CDER landscape analysis of NAMs submitted over ~15 years shows that, notably, human organ-on-a-chip/MPS were rarely submitted despite high external interest, suggesting a maturity/standardization gap between “promising tech” and “regulatory-ready evidence packages,” (5) indicating that next-generation complex models are still early in regulatory penetration.

To ensure consistency, existing ICH non-clinical guidelines and qualification procedures should be systematically reviewed and updated to explicitly reflect the acceptance of scientifically validated non-animal NAMs. Without such alignment, legacy language presuming animal data as the default standard may create contradictory messaging and regulatory uncertainty. Clear guidance is essential to support predictable, risk-based integration of NAM-derived data into regulatory decision-making. Beyond guideline updates, regulatory qualification procedures themselves require parallel evolution to support NAM adoption. The divergent approaches—EMA’s formal qualification opinion pathway versus FDA’s case-by-case acceptance—create procedural uncertainty and inconsistent interpretation of NAM acceptability across jurisdictions. As the industry transitions toward targeted replacement and substantial reduction of animal studies, harmonized qualification procedures are essential to establish transparent, standardized criteria for NAM acceptance, define timelines for qualification decisions, and clarify expectations regarding context of use, applicability domain, and performance standards. Such procedural alignment would reduce regulatory uncertainty, enable more efficient global development strategies, and support the transition from selective implementation toward broader, confidence-based NAM integration in non-clinical safety assessment. In summary, the most common regulatory-facing NAM evidence remains *in silico* + *in vitro*, while complex human-relevant platforms (e.g., organ-on-a-chip/MPS) are not yet broadly represented in submissions, reflecting remaining needs in standardization, performance characterization, and COU clarity. The state of the industry is best described as transitioning from proof-of-concept to selective implementation. NAMs are widely used and increasingly influential, but not yet sufficient for the entire replacement of animal testing in regulatory safety assessment. The likely “next phase” is not an abrupt shift to animal-free development, but a development toward targeted replacement, substantial reduction, and potentially more frequent single-species *in vivo* strategies, enabled by NAMs that are qualified for specific COU, operated under robust quality systems, and integrated transparently under a WoE framework. There is a growing need to develop robust models and methodologies capable of predicting relevant toxicological and pharmacological endpoints. The large volume and heterogeneity of data generated by advanced *in vitro* and NAM platforms can make comprehensive evaluation challenging for both drug developers and regulatory agencies, especially when data quality and standardization are variable. A recent draft guidance issued by the FDA/CDER (2026) is already heading in the right direction, providing additional direction on the validation and qualification of NAMs, emphasizing the definition of context of use and fit-for-purpose to support their integration into drug development and regulatory submission packages for decision-making (13). Progress reported against the objectives of the FDA’s 2025 roadmap (7) suggests that these initiatives are already yielding measurable regulatory impact (42). Key outputs include clarification of the qualification pathway for drug development tools – supported by more than 16 active submissions to the ISTAND Pilot Program (38), as well as the release of guidance for monoclonal antibodies (34), a liver-on-chip protocol, and a published 15-year analysis of new approach methodologies (NAMs) (5).
